# μ_3_-Chlorido-μ_2_-chlorido-(μ_3_-pyrrolidine-1-carbo­dithio­ato-κ^4^ *S*:*S*,*S*′:*S*′)tris­[(tri­ethyl­phosphane-κ*P*)copper(I)]: crystal structure and Hirshfeld surface analysis

**DOI:** 10.1107/S2056989017005382

**Published:** 2017-04-18

**Authors:** Yi Jiun Tan, Chien Ing Yeo, Nathan R. Halcovitch, Mukesh M. Jotani, Edward R. T. Tiekink

**Affiliations:** a Research Centre for Crystalline Materials, School of Science and Technology, Sunway University, 47500 Bandar Sunway, Selangor Darul Ehsan, Malaysia; b Department of Chemistry, Lancaster University, Lancaster LA1 4YB, United Kingdom; c Department of Physics, Bhavan’s Sheth R. A. College of Science, Ahmedabad, Gujarat 380001, India

**Keywords:** crystal structure, copper(I), di­thio­carbamate, Hirshfeld surface analysis

## Abstract

The title trinuclear compound, [Cu_3_(C_5_H_8_NS_2_)Cl_2_(C_6_H_15_P)_3_], has the di­thio­carbamate ligand symmetrically chelating one Cu^I^ atom and each of the S atoms bridging to another Cu^I^ atom. Both chloride ligands are bridging, one being μ_3_- and the other μ_2_-bridging. Each Et_3_P ligand occupies a terminal position. Two of the Cu^I^ atoms exist within Cl_2_PS donor sets and the third is based on a ClPS_2_ donor set, with each coordination geometry based on a distorted tetra­hedron. The constituents defining the core of the mol­ecule, *i.e.* Cu_3_Cl_2_S_2_, occupy seven corners of a distorted cube. In the crystal, linear supra­molecular chains along the *c* axis are formed *via* phosphane–methyl­ene-C—H⋯Cl and pyrrolidine–methyl­ene-C—H⋯π(chelate) inter­actions, and these chains pack without directional inter­actions between them. An analysis of the Hirshfeld surface points to the predominance of H atoms at the surface, *i.e.* contributing 86.6% to the surface, and also highlights the presence of C—H⋯π(chelate) inter­actions.

## Abstract 2

The di­thio­carbamate ligand chelates one Cu^I^ atom and each S atom bridges a second Cu^I^ atom and thus, is tetra­coordinate. The core of the mol­ecule comprises Cu_3_Cl_2_S_2_ and defines seven corners of a distorted cube.

## Chemical context   

Recent studies have highlighted the potential of ternary coinage metal phosphane/di­thio­carbamates as anti-microbial agents. Motivated by the quite significant activity exhibited by *R*_3_PAu(S_2_CN*RR*′), *R*, *R*′ = alk­yl/aryl (Sim *et al.*, 2014 ▸; Chen *et al.*, 2016 ▸), lower congeners, *i.e*. (Ph_3_P)_2_ *M*(S_2_CN*RR*′), *M* = Cu^I^ and Ag^I^, were investigated and shown to be also potent in this context (Jamaludin *et al.*, 2016 ▸). A prominent lead compound, Et_3_PAu(S_2_CNEt_2_), was shown to possess broad-range activity against Gram-positive and Gram-negative bacteria and, notably, was also bactericidal against methicillin-resistant *Staphylococcus aureus* (MRSA) (Chen *et al.*, 2016 ▸). Given that Et_3_PAu(S_2_CNEt_2_) exhibited the most exciting potential amongst the phosphanegold di­thio­carbamates, it was thought of inter­est to extend the chemistry/biological investigations of (*R*_3_P)_2_ *M*(S_2_CN*RR*’), *M* = Cu^I^ and Ag^I^, to include trialkyl­phosphane species. It was during these studies that the title compound, (I), was isolated as an incomplete reaction product from the 1:2:1 reaction between CuCl, Et_3_P and NH_4_[S_2_CN(CH_2_)_4_]. Herein, the crystal and mol­ecular structures of (I) are described along with a detailed analysis of the Hirshfeld surface.
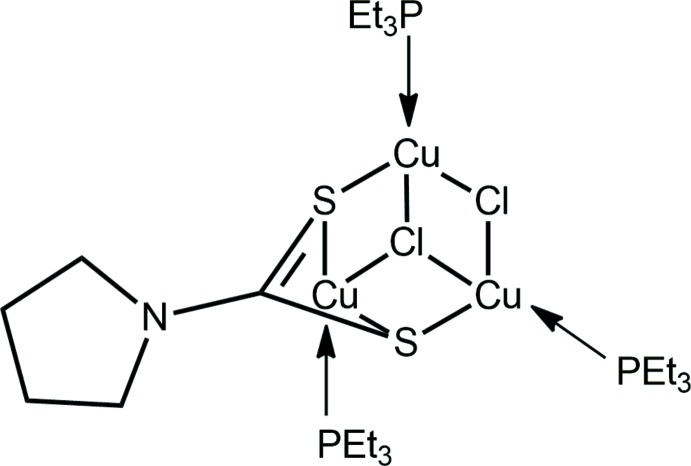


## Structural commentary   

**Figure 1:**
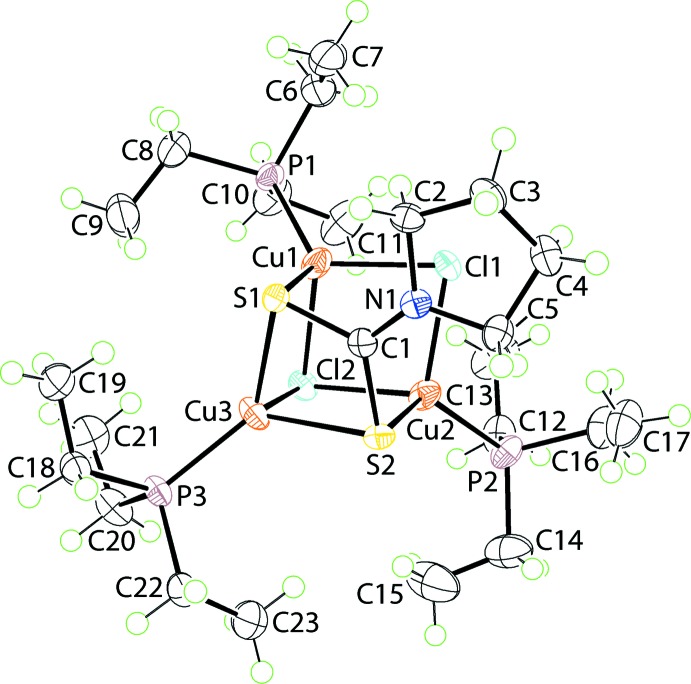
The mol­ecular structure of (I), showing the atom-labelling scheme and displacement ellipsoids at the 70% probability level.

**Figure 2:**
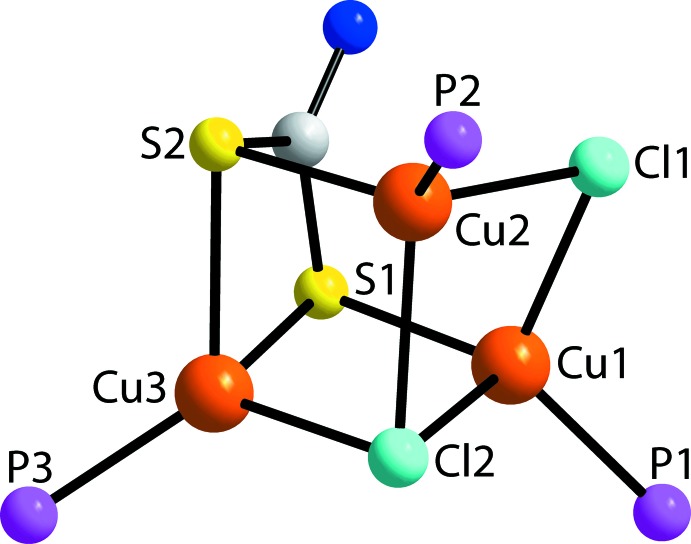
The mol­ecular core in (I) highlighting the ‘incomplete cube’.

The mol­ecular structure of (I), Fig. 1 ▸, represents a neutral, trinuclear Cu^I^ complex comprising three monodentate phosphane ligands, two chlorido anions, one μ_3_- and the other μ_2_-bridg­ing, and a di­thio­carbamate ligand. The latter is tetra-coordinat­ing, chelating the Cu3 atom, and each sulfur atom also bridges another Cu^I^ atom. As highlighted in Fig. 2 ▸, the Cu_3_Cl_2_S_2_ atoms of the core occupy the corners of a distorted cube with the putative eighth position being occupied by the quaternary-carbon atom of the di­thio­carbamate ligand. As listed in Table 1 ▸, there are systematic trends in the Cu—donor-atom bond lengths. To a first approximation, the Cu—P bond lengths are about the same. As anti­cipated for the Cu1 and Cu2 atoms, the Cu—Cl bond lengths involving the μ_3_-chlorido ligand are systematically longer than those formed with the μ_2_-chlorido ligand. Despite being chelated by the di­thio­carbamate ligand, the Cu3 atom forms longer Cu—S bond lengths than do the Cu1 and Cu2 atoms, an observation correlated with the presence of two electronegative chloride anions in the donor sets for the latter.

The coordination geometries for the Cu1 and Cu2 atoms are based on Cl_2_PS donor sets while that of Cu3 is based on a ClPS_2_ donor set, Table 1 ▸. While being based on tetra­hedra, the coordination geometries exhibit wide ranges of angles subtended at the copper atoms, *i.e*. 30, 28 and 53°, respectively. The wider range of angles about the Cu3 atom can be traced, in part, to the acute angle subtended by the di­thio­carbamate ligand. A measure of the geometry defined by a four-atom donor set is τ_4_ (Yang *et al.*, 2007 ▸). Based on this index, τ_4_ values of 1 and 0 are computed for ideal tetra­hedral and square-planar geometries, respectively. The τ_4_ values calculated for the Cu1–Cu3 atoms in (I) are 0.84, 0.86 and 0.78, respectively, *i.e*. consistent with distortions from tetra­hedral geometries.

Reflecting the near equivalence in the pairs of Cu—S1 and Cu—S2 bonds, the associated C—S bond lengths are equal within experimental error, Table 1 ▸. Finally, the pyrrolidine ring is twisted about the C3—C4 bond.

## Supra­molecular features   

**Figure 3:**
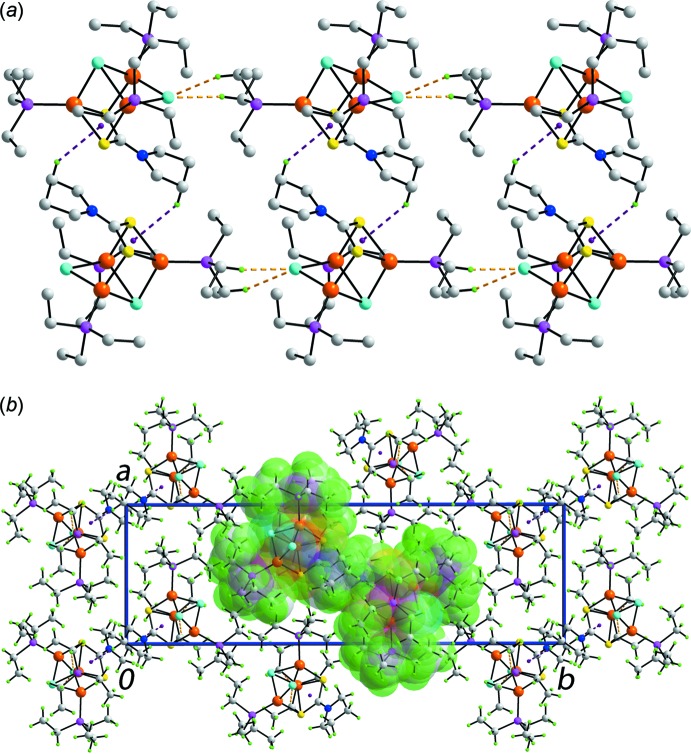
The mol­ecular packing in (I): (*a*) linear supra­molecular chain mediated by methyl­ene-C—H⋯Cl (orange dashed lines) and methyl­ene-C—H⋯π(chelate) (blue) inter­actions aligned along the *c* axis and (*b*) view of the unit-cell contents in projection down the *c* axis. One chain is highlighted in space-filling mode.

The key feature of the mol­ecular packing in (I) is the formation of linear supra­molecular chains along the *c* axis, Fig. 3 ▸ *a* and Table 2 ▸. The μ_2_-chlorido ligand accepts two phosphane-methyl­ene-C—H⋯Cl type inter­actions to form a linear chain. Centrosymmetrically related chains are connected *via* pyrrolidine–methyl­ene-C—H⋯π(chelate) inter­actions where the chelate ring is defined by the Cu1,S1,S2,C1 atoms. Such C—H⋯π(chelate) inter­actions are now well established in di­thio­carbamate structural chemistry (Tiekink & Zukerman-Schpector, 2011 ▸) and are gaining greater recognition in coordination chemistry (Tiekink, 2017 ▸). The supra­molecular chains pack in the crystal with no directional inter­actions between them, Fig. 3 ▸ *b*.

## Hirshfeld surface analysis   

**Figure 4:**
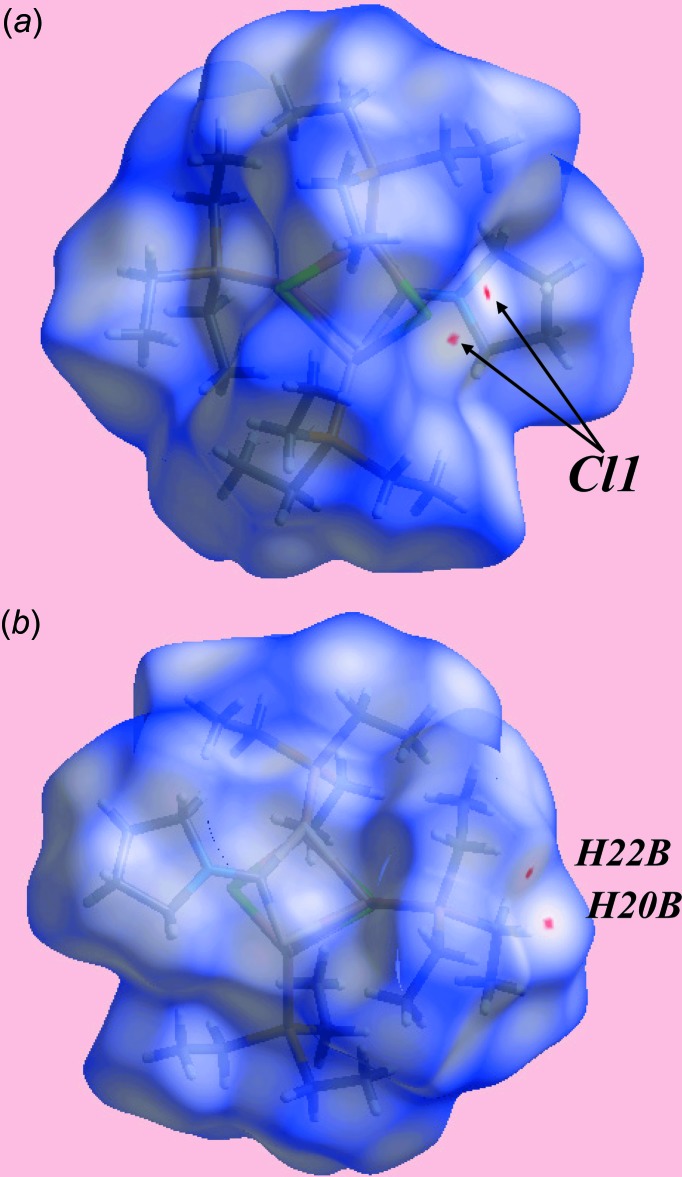
Two views of the Hirshfeld surface for (I) mapped over *d*_norm_ over the range −0.016 to 1.529 au.

**Figure 5:**
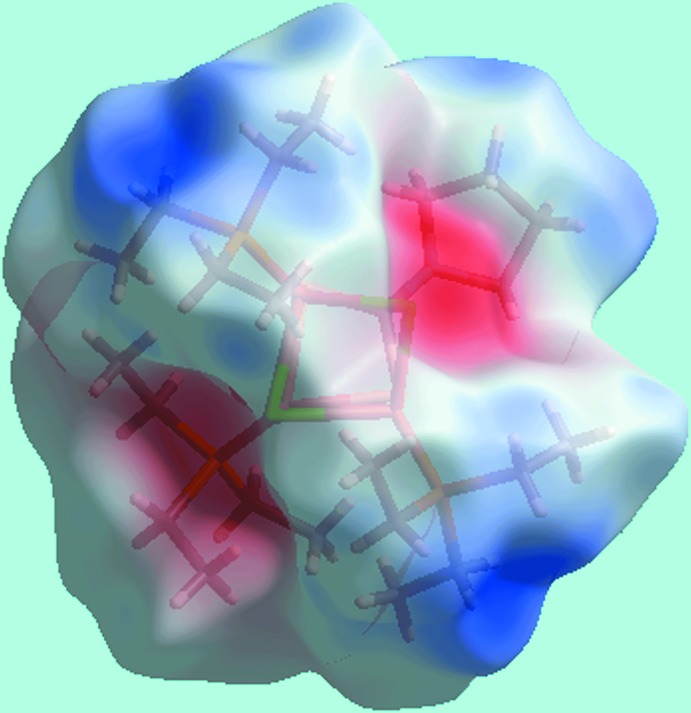
A view of the Hirshfeld surface for (I) mapped over the calculated electrostatic potential in the range −0.071 to 0.030 au. The red and blue regions represent negative and positive electrostatic potentials, respectively.

**Figure 6:**
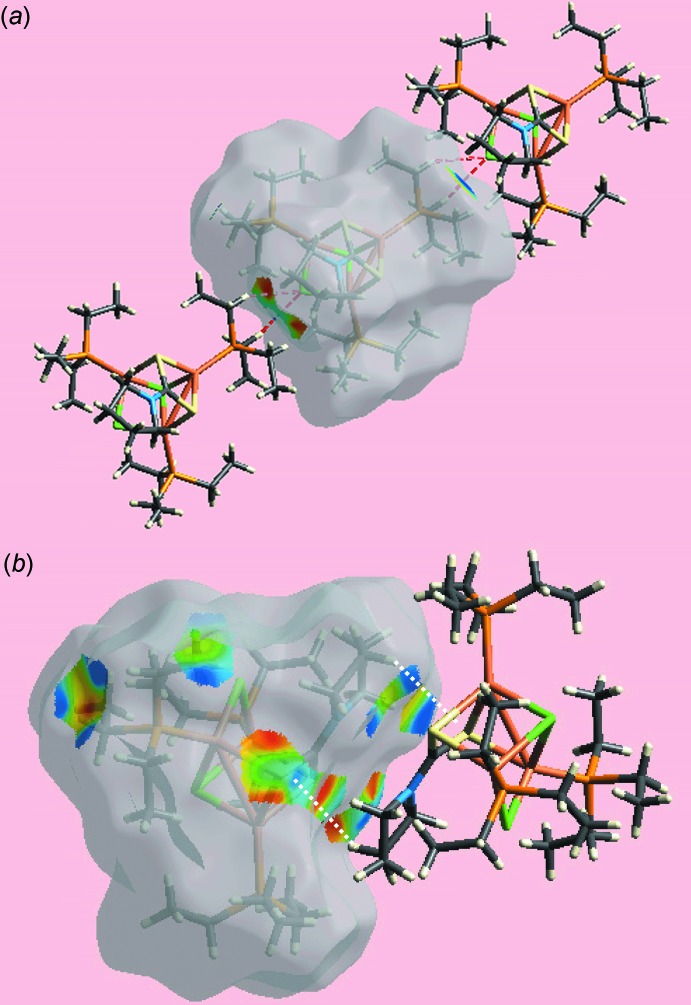
Views of Hirshfeld surface for a reference mol­ecule in (I) mapped over the shape-index property highlighting the: (*a*) C—H⋯Cl inter­actions as red dashed lines and (*b*) C—H⋯π(chelate) inter­actions as white dashed lines

The Hirshfeld surface analysis of (I) was performed in accord with a recent study of a related di­thio­carbamate complex (Jotani *et al.*, 2016 ▸). The presence of tiny red spots near the Cl1 and methyl­ene-H20*B* and H22*B* atoms on the Hirshfeld surfaces mapped over *d*_norm_ in Fig. 4 ▸ is indicative of the double-acceptor (C—H)_2_⋯Cl inter­action. In the view of the Hirshfeld surface mapped over the calculated electrostatic potential in Fig. 5 ▸, the light-blue and pale-red regions around the electropositive and electronegative atoms result from the polarization of charges about the donors and acceptors, respectively, of the inter­molecular inter­actions. The immediate environments about a reference mol­ecule within the shape-index-mapped Hirshfeld surfaces in Fig. 6 ▸ *a* and *b* highlight the inter­molecular C—H⋯Cl and C—H⋯π(chelate) inter­actions, respectively.

**Figure 7:**
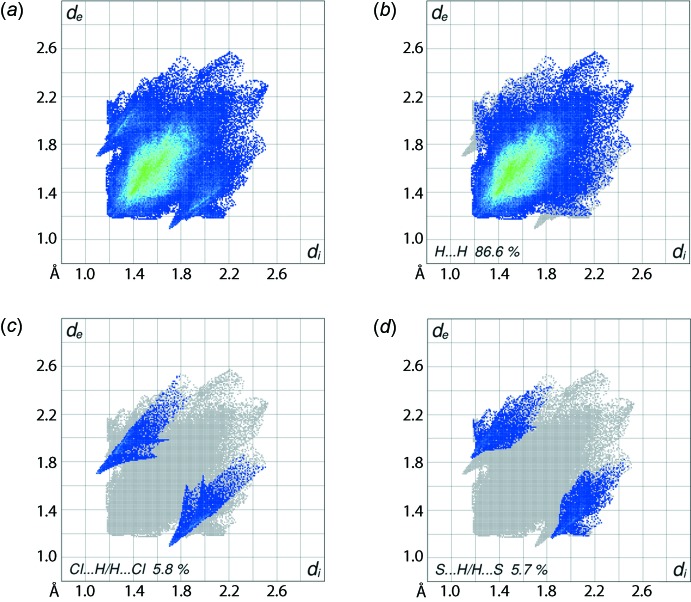
(*a*) The full two-dimensional fingerprint plot for (I) and fingerprint plots delineated into (*b*) H⋯H, (*c*) Cl⋯H/H⋯Cl and (*d*) S⋯H/H⋯S contacts.

The two-dimensional fingerprint plots for (I), *i.e*. the overall, Fig. 7 ▸ *a*, and those delineated into H⋯H, Cl⋯H/H⋯Cl and S⋯H/H⋯S contacts (McKinnon *et al.*, 2007 ▸) in Fig. 7 ▸ *b*–*d*, respectively, provide further information on the inter­molecular inter­actions present in the crystal. It is evident from the fingerprint plot delineated into H⋯H contacts, Fig. 7 ▸ *b*, that the hydrogen atoms of the tri­ethyl­phosphane and pyrrolidine ligands make the greatest contribution, *i.e*. 86.6%, to the Hirshfeld surface, but at distances greater than the sum of the van der Waals radii. The pair of tips at *d*_e_ + *d*_i_ ∼ 2.8 Å in the arrow-like distribution of points in the plot for Cl⋯H/H⋯Cl contacts, Fig. 7 ▸ *c*, represent the inter­molecular C—H⋯Cl inter­actions. A pair of short spikes at *d*_e_ + *d*_i_ ∼ 3.0 Å in the S⋯H/H⋯S delineated plot, Fig. 7 ▸ *d*, and the 5.8% contribution to Hirshfeld surfaces along with the small but significant contributions from C⋯H/H⋯C and Cu⋯H/H⋯Cu contacts, Table 3 ▸, to the Hirshfeld surface are all indicative of the C—H⋯π(chelate) inter­action, Fig. 3 ▸ *a* and Table 2 ▸. The small contributions from the other inter­atomic contacts, namely N⋯H/H⋯N and C⋯N/N⋯C, have little effect on the packing of the crystal.

## Database survey   

The isolated Cu_3_(μ_3_-Cl)(μ_2_-Cl)S_2_ core observed in (I) appears to be rare in the literature, being structurally observed only in one other structure with general formula, *M*_3_(μ_3_-*X*)(μ_2_-*X*)S_2_, incidentally, a di­thio­carbamate complex. Thus, in the Ru^II^ species, Ru_3_(CO)_3_(S_2_CNEt_2_)_4_Cl_2_, a discrete Ru_3_(μ_3_-Cl)(μ_2_-Cl)S_2_ core is found but where the μ_2_-S sulfur atoms are derived from four di­thio­carbamate ligands and each Ru^II^ atom is coordinated by two additional sulfur donor atoms leading to *trans*-RuCClS_4_ octa­hedral coordination geometries (Raston & White, 1975 ▸). While other structures are known with the specified core, the core is embedded within higher nuclearity clusters or in coordination polymers.

There are twenty crystal structure containing copper with di­thio­carbamate and phosphane ligands in the crystallographic literature (Groom *et al.*, 2016 ▸). The majority, *i.e*. 12 conform to the tetra­hedral CuP_2_S_2_ motif observed in the biologically active bis­(phosphane)copper(I) di­thio­carbamate compounds mentioned in the *Chemical Context* (Jamaludin *et al.* 2016 ▸; Tan *et al.*, 2016 ▸). Similar coordination geometries are found in two binuclear structures with bis­(di­thio­carbamate) ligands, as exemplified in (Ph_3_P)_2_CuS_2_CN(CH_2_CH_2_)_2_NCS_2_Cu(PPh_3_)_2_ (Kumar *et al.*, 2009 ▸). There are two related complexes but with a 1:1:1 ratio of copper, di­thio­carbamate and phosphane, as exemplified by [Et_3_PCu(S_2_CNEt_2_)]_2_ (Afzaal *et al.*, 2011 ▸). One of the remaining structures is neutral and octa­nuclear with formula (Ph_3_P)_4_Cu_8_(μ_4_-SC_6_H_4_Br-4)_4_(μ_2_-SC_6_H_4_Br-4)_2_(S_2_CNMe_2_)_2_·MeO(CH_2_)_2_OMe (Langer *et al.*, 2009 ▸). Here, each sulfur atom of the di­thio­carbamate ligand bridges two different Cu^I^ atoms. The common feature of the remaining three structures is that they are charged and feature bidentate phosphane ligands. The simplest of these is formulated as [(dppm)_2_Cu_2_(S_2_CNMe_2_)][ClO_4_]_2_·EtOH·0.25H_2_O where the di­thio­carbamate ligand is bidentate bridging as is the dppm ligand (Huang & Situ, 2003 ▸); dppm = Ph_2_PCH_2_PPh_2_. In the trinuclear mono-cation {(dppm)_3_Cu_3_(μ_3_-I)[S_2_CN(CH_2_Ph)CH_2_(2-thien­yl)]}I, the di­thio­carbamate ligand bridges two Cu^I^ atoms and simultaneously coordinates a third Cu^I^ atom *via* one of the sulfur atoms only (Rajput *et al.*, 2015 ▸). The final structure to be described is related to the former whereby one bis­(phosphane) ligand has been replaced by a di­thio­carbamate ligand with the ejection of the μ_3_-iodido species, *i.e*. {(dppf)_2_Cu_3_[S_2_CN(CH_2_Ph)CH_2_Fc]_2_}PF_6_·CHCl_3_ (Kishore *et al.*, 2016 ▸); dppf = Ph_2_P(η^5^-C_5_H_4_)Fe(η^5^-C_5_H_4_)PPh_2_ and Fc is (η^5^-C_5_H_4_)Fe(η^5^-C_5_H_5_). In this structure, each di­thio­carbamate ligand is tri-coordinate, binding to three different Cu^I^ atoms. From the foregoing, it is obvious there is considerable structural variability in these systems arising in part from the ability of the di­thio­carbamate ligands to adopt quite diverse coordination modes.

## Synthesis and crystallization   

Complex (I) is an unexpected product from the *in situ* reaction of CuCl, Et_3_P, and NH_4_[S_2_CN(CH_2_)_4_] in a 1:2:1 ratio. The preparation was as follows: NH_4_[S_2_CN(CH_2_)_4_] (Sigma–Aldrich, 0.5 mmol, 0.082 g) dissolved in iso­propanol (5 ml) was added to an iso­propanol solution (5 ml) of CuCl (Sigma–Aldrich, 0.5 mmol, 0.05 g) at room temperature. Then, a THF solution of Et_3_P (Sigma–Aldrich; 1 ml (= 0.118 g), 1.0 mmol) was added to the reaction mixture followed by stirring for 2 h. The resulting mixture was filtered, diluted with hexane (2 ml) and mixed well. The mixture was left for evaporation at 227 K. A small number of yellow crystals of (I) were obtained after 5 d. Yield: 0.0095 g (4.26%), m.p. 330.8 K. IR (cm^−1^): 1429(*s*) *v*(C—N); 1045(*m*), 993(*m*) *v*(C—S).

## Refinement   

Crystal data, data collection and structure refinement details are summarized in Table 4 ▸. Carbon-bound H atoms were placed in calculated positions (C—H = 0.98–0.99 Å) and were included in the refinement in the riding model approximation, with *U*_iso_(H) set to 1.2–1.5*U*_eq_(C).

## Supplementary Material

Crystal structure: contains datablock(s) I, global. DOI: 10.1107/S2056989017005382/hb7670sup1.cif

Structure factors: contains datablock(s) I. DOI: 10.1107/S2056989017005382/hb7670Isup2.hkl

CCDC reference: 1543298

Additional supporting information:  crystallographic information; 3D view; checkCIF report

## Figures and Tables

**Table 1:** Selected geometric parameters (Å, °)

Cu1—Cl1	2.3474 (5)	Cu2—P2	2.2018 (6)
Cu1—Cl2	2.5809 (5)	Cu3—Cl2	2.3912 (5)
Cu1—S1	2.3282 (5)	Cu3—S1	2.4002 (5)
Cu1—P1	2.1936 (5)	Cu3—S2	2.4939 (5)
Cu2—Cl1	2.3640 (5)	Cu3—P3	2.1841 (5)
Cu2—Cl2	2.5324 (5)	S1—C1	1.7367 (19)
Cu2—S2	2.3556 (5)	S2—C1	1.7330 (19)
			
Cl1—Cu1—Cl2	96.188 (18)	Cl2—Cu2—S2	97.904 (18)
Cl1—Cu1—S1	104.585 (19)	Cl2—Cu2—P2	112.82 (2)
Cl1—Cu1—P1	115.51 (2)	S2—Cu2—P2	124.87 (2)
Cl2—Cu1—S1	100.954 (18)	Cl2—Cu3—S1	104.566 (18)
Cl2—Cu1—P1	108.90 (2)	Cl2—Cu3—S2	98.030 (18)
S1—Cu1—P1	125.81 (2)	Cl2—Cu3—P3	118.56 (2)
Cl1—Cu2—Cl2	97.080 (18)	S1—Cu3—S2	74.935 (17)
Cl1—Cu2—S2	106.406 (19)	S1—Cu3—P3	127.39 (2)
Cl1—Cu2—P2	113.35 (2)	S2—Cu3—P3	123.04 (2)

**Table 2:** Hydrogen-bond geometry (Å, °) *Cg*1 is the centroid of the (Cu,S1,S2,C1) chelate ring.

*D*—H⋯*A*	*D*—H	H⋯*A*	*D*⋯*A*	*D*—H⋯*A*
C20—H20*B*⋯Cl1^i^	0.99	2.81	3.722 (2)	154
C22—H22*B*⋯Cl1^i^	0.99	2.80	3.720 (2)	154
C3—H3*B*⋯*Cg*1^ii^	0.99	2.83	3.705 (2)	148

Symmetry codes: (i) 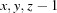; (ii) 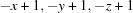.

**Table 3:** Percentage contribution of inter­atomic contacts to the Hirshfeld surface for (I)

Contact	percentage contribution
H⋯H	86.6
Cl⋯H/H⋯Cl	5.8
S⋯H/H⋯S	5.7
C⋯H/H⋯C	1.1
Cu⋯H/H⋯Cu	0.4
N⋯H/H⋯N	0.3
C⋯N / N⋯C	0.1

**Table 4:** Experimental details

Crystal data	
Chemical formula	[Cu_3_(C_5_H_8_NS_2_)Cl_2_(C_6_H_15_P)_3_]
*M*_r_	762.21
Crystal system, space group	Monoclinic, *P*2_1_/*n*
Temperature (K)	100
*a*, *b*, *c* (Å)	10.6489 (2), 31.7578 (4), 10.7212 (2)
β (°)	108.607 (2)
*V* (Å^3^)	3436.24 (11)
*Z*	4
Radiation type	Cu *K*α
μ (mm^−1^)	6.14
Crystal size (mm)	0.20 × 0.09 × 0.07

Data collection	
Diffractometer	Agilent SuperNova, Dual, Cu at zero, AtlasS2
Absorption correction	Multi-scan (*CrysAlis PRO*; Rigaku Oxford Diffraction, 2015 ▸)
*T*_min_, *T*_max_	0.684, 1.000
No. of measured, independent and observed [*I* > 2σ(*I*)] reflections	34566, 7186, 6699
*R*_int_	0.027
(sin θ/λ)_max_ (Å^−1^)	0.631

Refinement	
*R*[*F* ^2^ > 2σ(*F* ^2^)], *wR*(*F* ^2^), *S*	0.028, 0.073, 1.04
No. of reflections	7186
No. of parameters	316
H-atom treatment	H-atom parameters constrained
Δρ_max_, Δρ_min_ (e Å^−3^)	1.50, −0.79

Computer programs: *CrysAlis PRO* (Rigaku Oxford Diffraction, 2015 ▸), *SHELXS* (Sheldrick, 2008 ▸), *SHELXL2014* (Sheldrick, 2015 ▸), *ORTEP-3 for Windows* (Farrugia, 2012 ▸), *DIAMOND* (Brandenburg, 2006 ▸) and *publCIF* (Westrip, 2010 ▸).

## supplementary crystallographic information

### Crystal data

**Table:**

[Cu_3_(C_5_H_8_NS_2_)Cl_2_(C_6_H_15_P)_3_]	*F*(000) = 1584
*M**_r_* = 762.21	*D*_x_ = 1.473 Mg m^−^^3^
Monoclinic, *P*2_1_/*n*	Cu *K*α radiation, λ = 1.54184 Å
*a* = 10.6489 (2) Å	Cell parameters from 16968 reflections
*b* = 31.7578 (4) Å	θ = 2.8–76.3°
*c* = 10.7212 (2) Å	µ = 6.14 mm^−^^1^
β = 108.607 (2)°	*T* = 100 K
*V* = 3436.24 (11) Å^3^	Prism, yellow
*Z* = 4	0.20 × 0.09 × 0.07 mm

### Data collection

**Table:**

Agilent SuperNova, Dual, Cu at zero, AtlasS2 diffractometer	7186 independent reflections
Radiation source: micro-focus sealed X-ray tube	6699 reflections with *I* > 2σ(*I*)
Mirror monochromator	*R*_int_ = 0.027
Detector resolution: 10.4607 pixels mm^-1^	θ_max_ = 76.6°, θ_min_ = 2.8°
ω scans	*h* = −13→12
Absorption correction: multi-scan (CrysAlisPro; Rigaku Oxford Diffraction, 2015)	*k* = −39→29
*T*_min_ = 0.684, *T*_max_ = 1.000	*l* = −13→13
34566 measured reflections	

### Refinement

**Table:**

Refinement on *F*^2^	0 restraints
Least-squares matrix: full	Hydrogen site location: inferred from neighbouring sites
*R*[*F*^2^ > 2σ(*F*^2^)] = 0.028	H-atom parameters constrained
*wR*(*F*^2^) = 0.073	*w* = 1/[σ^2^(*F*_o_^2^) + (0.0334*P*)^2^ + 3.4591*P*] where *P* = (*F*_o_^2^ + 2*F*_c_^2^)/3
*S* = 1.04	(Δ/σ)_max_ = 0.002
7186 reflections	Δρ_max_ = 1.50 e Å^−^^3^
316 parameters	Δρ_min_ = −0.79 e Å^−^^3^

### Special details

**Table:**

Geometry. All esds (except the esd in the dihedral angle between two l.s. planes) are estimated using the full covariance matrix. The cell esds are taken into account individually in the estimation of esds in distances, angles and torsion angles; correlations between esds in cell parameters are only used when they are defined by crystal symmetry. An approximate (isotropic) treatment of cell esds is used for estimating esds involving l.s. planes.

### Fractional atomic coordinates and isotropic or equivalent isotropic displacement parameters (Å^2^)

**Table:**

	*x*	*y*	*z*	*U*_iso_*/*U*_eq_	
Cu1	0.15403 (3)	0.60427 (2)	0.46213 (3)	0.02150 (8)	
Cu2	0.41213 (3)	0.65484 (2)	0.54323 (3)	0.02075 (8)	
Cu3	0.29958 (3)	0.60979 (2)	0.26251 (3)	0.02196 (8)	
Cl1	0.29973 (4)	0.62046 (2)	0.67210 (4)	0.01860 (9)	
Cl2	0.21102 (4)	0.66892 (2)	0.34453 (4)	0.01933 (9)	
S1	0.26010 (4)	0.55091 (2)	0.38531 (4)	0.01620 (9)	
S2	0.50822 (4)	0.60146 (2)	0.45081 (4)	0.01641 (9)	
P1	−0.05739 (5)	0.60624 (2)	0.44227 (5)	0.01844 (10)	
P2	0.51349 (5)	0.71293 (2)	0.63413 (5)	0.02172 (11)	
P3	0.29464 (5)	0.61039 (2)	0.05729 (5)	0.01832 (10)	
N1	0.46529 (16)	0.53980 (5)	0.60120 (15)	0.0162 (3)	
C1	0.41813 (18)	0.56133 (6)	0.49156 (18)	0.0152 (3)	
C2	0.38664 (19)	0.50826 (6)	0.64599 (19)	0.0190 (4)	
H2A	0.3621	0.4843	0.5840	0.023*	
H2B	0.3049	0.5210	0.6546	0.023*	
C3	0.4799 (2)	0.49415 (6)	0.78003 (19)	0.0222 (4)	
H3A	0.4297	0.4867	0.8403	0.027*	
H3B	0.5338	0.4697	0.7709	0.027*	
C4	0.5667 (2)	0.53283 (6)	0.82932 (19)	0.0238 (4)	
H4A	0.5202	0.5540	0.8665	0.029*	
H4B	0.6510	0.5250	0.8972	0.029*	
C5	0.59098 (19)	0.54950 (6)	0.70556 (19)	0.0201 (4)	
H5A	0.6084	0.5802	0.7119	0.024*	
H5B	0.6666	0.5349	0.6896	0.024*	
C6	−0.0943 (2)	0.58600 (9)	0.5866 (2)	0.0350 (5)	
H6A	−0.0571	0.6055	0.6612	0.042*	
H6B	−0.1916	0.5853	0.5674	0.042*	
C7	−0.0388 (3)	0.54232 (9)	0.6268 (3)	0.0417 (6)	
H7A	−0.0801	0.5224	0.5558	0.063*	
H7B	−0.0576	0.5337	0.7068	0.063*	
H7C	0.0572	0.5426	0.6437	0.063*	
C8	−0.1738 (2)	0.57717 (7)	0.3059 (2)	0.0285 (4)	
H8A	−0.1613	0.5466	0.3233	0.034*	
H8B	−0.2656	0.5844	0.3014	0.034*	
C9	−0.1549 (3)	0.58705 (9)	0.1739 (2)	0.0379 (6)	
H9A	−0.1690	0.6172	0.1553	0.057*	
H9B	−0.2189	0.5709	0.1043	0.057*	
H9C	−0.0647	0.5794	0.1772	0.057*	
C10	−0.1319 (2)	0.65879 (8)	0.4228 (3)	0.0332 (5)	
H10A	−0.1436	0.6691	0.3326	0.040*	
H10B	−0.2207	0.6569	0.4335	0.040*	
C11	−0.0480 (3)	0.69041 (8)	0.5220 (3)	0.0432 (6)	
H11A	−0.0424	0.6817	0.6112	0.065*	
H11B	−0.0890	0.7183	0.5040	0.065*	
H11C	0.0412	0.6915	0.5144	0.065*	
C12	0.4059 (2)	0.75778 (7)	0.6313 (2)	0.0287 (5)	
H12A	0.4587	0.7807	0.6861	0.034*	
H12B	0.3690	0.7683	0.5400	0.034*	
C13	0.2925 (3)	0.74600 (9)	0.6827 (3)	0.0435 (6)	
H13A	0.2441	0.7219	0.6328	0.065*	
H13B	0.2322	0.7700	0.6723	0.065*	
H13C	0.3284	0.7385	0.7761	0.065*	
C14	0.6344 (3)	0.73513 (8)	0.5657 (3)	0.0387 (6)	
H14A	0.6573	0.7640	0.5999	0.046*	
H14B	0.7163	0.7180	0.5937	0.046*	
C15	0.5811 (4)	0.73634 (9)	0.4171 (3)	0.0517 (8)	
H15A	0.5621	0.7076	0.3830	0.078*	
H15B	0.6471	0.7491	0.3828	0.078*	
H15C	0.4995	0.7531	0.3893	0.078*	
C16	0.6077 (2)	0.70895 (7)	0.8114 (2)	0.0322 (5)	
H16A	0.6675	0.7335	0.8377	0.039*	
H16B	0.5451	0.7098	0.8626	0.039*	
C17	0.6886 (3)	0.66909 (9)	0.8443 (3)	0.0393 (6)	
H17A	0.6292	0.6447	0.8289	0.059*	
H17B	0.7439	0.6697	0.9369	0.059*	
H17C	0.7454	0.6669	0.7884	0.059*	
C18	0.2471 (2)	0.56150 (6)	−0.03875 (19)	0.0223 (4)	
H18A	0.2167	0.5687	−0.1335	0.027*	
H18B	0.3265	0.5434	−0.0219	0.027*	
C19	0.1383 (2)	0.53638 (7)	−0.0080 (2)	0.0291 (5)	
H19A	0.1670	0.5292	0.0858	0.044*	
H19B	0.1209	0.5105	−0.0604	0.044*	
H19C	0.0573	0.5533	−0.0297	0.044*	
C20	0.1873 (2)	0.65065 (7)	−0.0452 (2)	0.0262 (4)	
H20A	0.2228	0.6788	−0.0128	0.031*	
H20B	0.1890	0.6478	−0.1366	0.031*	
C21	0.0443 (2)	0.64775 (8)	−0.0457 (2)	0.0336 (5)	
H21A	0.0058	0.6210	−0.0855	0.050*	
H21B	−0.0069	0.6712	−0.0969	0.050*	
H21C	0.0422	0.6492	0.0448	0.050*	
C22	0.4553 (2)	0.62219 (7)	0.0359 (2)	0.0237 (4)	
H22A	0.5156	0.5980	0.0675	0.028*	
H22B	0.4427	0.6259	−0.0590	0.028*	
C23	0.5198 (3)	0.66167 (8)	0.1099 (2)	0.0358 (5)	
H23A	0.4631	0.6861	0.0754	0.054*	
H23B	0.6064	0.6660	0.0981	0.054*	
H23C	0.5312	0.6583	0.2038	0.054*	

### Atomic displacement parameters (Å^2^)

**Table:**

	*U*^11^	*U*^22^	*U*^33^	*U*^12^	*U*^13^	*U*^23^
Cu1	0.01310 (14)	0.02656 (16)	0.02473 (15)	−0.00123 (11)	0.00588 (11)	−0.00501 (12)
Cu2	0.02180 (15)	0.01514 (14)	0.02611 (15)	−0.00383 (11)	0.00876 (12)	−0.00443 (11)
Cu3	0.03038 (17)	0.02277 (15)	0.01416 (14)	0.00065 (12)	0.00914 (12)	0.00068 (11)
Cl1	0.0220 (2)	0.0212 (2)	0.01358 (18)	0.00073 (16)	0.00701 (16)	−0.00123 (15)
Cl2	0.0218 (2)	0.0185 (2)	0.0179 (2)	0.00308 (16)	0.00661 (17)	0.00183 (15)
S1	0.0173 (2)	0.0152 (2)	0.0159 (2)	−0.00239 (15)	0.00490 (16)	−0.00200 (15)
S2	0.0158 (2)	0.0155 (2)	0.0199 (2)	−0.00078 (15)	0.00855 (17)	−0.00004 (16)
P1	0.0128 (2)	0.0219 (2)	0.0203 (2)	−0.00112 (17)	0.00482 (18)	−0.00132 (18)
P2	0.0190 (2)	0.0146 (2)	0.0294 (3)	−0.00147 (17)	0.0047 (2)	−0.00271 (19)
P3	0.0240 (2)	0.0181 (2)	0.0136 (2)	0.00122 (18)	0.00702 (18)	−0.00037 (17)
N1	0.0171 (7)	0.0130 (7)	0.0189 (7)	0.0005 (6)	0.0064 (6)	−0.0003 (6)
C1	0.0172 (8)	0.0131 (8)	0.0169 (8)	0.0018 (6)	0.0078 (7)	−0.0028 (6)
C2	0.0225 (9)	0.0150 (9)	0.0210 (9)	0.0001 (7)	0.0088 (8)	0.0029 (7)
C3	0.0296 (11)	0.0186 (9)	0.0196 (9)	0.0032 (8)	0.0096 (8)	0.0026 (7)
C4	0.0299 (11)	0.0213 (10)	0.0177 (9)	0.0030 (8)	0.0038 (8)	−0.0001 (7)
C5	0.0190 (9)	0.0173 (9)	0.0212 (9)	0.0013 (7)	0.0024 (7)	0.0000 (7)
C6	0.0254 (11)	0.0531 (15)	0.0265 (11)	−0.0062 (10)	0.0082 (9)	0.0025 (10)
C7	0.0346 (13)	0.0498 (16)	0.0327 (12)	−0.0154 (11)	−0.0005 (10)	0.0135 (11)
C8	0.0230 (10)	0.0317 (11)	0.0286 (11)	−0.0046 (9)	0.0050 (8)	−0.0029 (9)
C9	0.0364 (13)	0.0504 (15)	0.0243 (11)	−0.0012 (11)	0.0062 (10)	−0.0042 (10)
C10	0.0245 (11)	0.0307 (12)	0.0426 (13)	0.0035 (9)	0.0083 (10)	−0.0036 (10)
C11	0.0314 (13)	0.0334 (13)	0.0651 (18)	−0.0017 (10)	0.0159 (12)	−0.0186 (12)
C12	0.0337 (12)	0.0184 (10)	0.0300 (11)	0.0038 (8)	0.0045 (9)	−0.0029 (8)
C13	0.0337 (13)	0.0317 (13)	0.0680 (18)	0.0024 (10)	0.0204 (13)	−0.0126 (12)
C14	0.0443 (14)	0.0300 (12)	0.0513 (15)	−0.0103 (10)	0.0286 (13)	−0.0079 (11)
C15	0.085 (2)	0.0332 (14)	0.0538 (17)	−0.0146 (14)	0.0455 (17)	−0.0111 (12)
C16	0.0313 (12)	0.0290 (11)	0.0310 (11)	−0.0019 (9)	0.0027 (9)	−0.0041 (9)
C17	0.0324 (13)	0.0386 (14)	0.0374 (13)	−0.0003 (10)	−0.0021 (10)	0.0007 (11)
C18	0.0281 (10)	0.0209 (9)	0.0181 (9)	−0.0013 (8)	0.0076 (8)	−0.0014 (7)
C19	0.0319 (12)	0.0264 (11)	0.0281 (11)	−0.0069 (9)	0.0084 (9)	−0.0022 (8)
C20	0.0341 (11)	0.0236 (10)	0.0232 (10)	0.0101 (8)	0.0123 (9)	0.0034 (8)
C21	0.0300 (12)	0.0345 (12)	0.0349 (12)	0.0096 (9)	0.0082 (10)	0.0020 (10)
C22	0.0269 (10)	0.0260 (10)	0.0185 (9)	−0.0022 (8)	0.0074 (8)	−0.0019 (8)
C23	0.0417 (14)	0.0367 (13)	0.0304 (12)	−0.0135 (11)	0.0132 (10)	−0.0083 (10)

### Geometric parameters (Å, º)

**Table:**

Cu1—Cl1	2.3474 (5)	C8—H8B	0.9900
Cu1—Cl2	2.5809 (5)	C9—H9A	0.9800
Cu1—S1	2.3282 (5)	C9—H9B	0.9800
Cu1—P1	2.1936 (5)	C9—H9C	0.9800
Cu2—Cl1	2.3640 (5)	C10—C11	1.527 (3)
Cu2—Cl2	2.5324 (5)	C10—H10A	0.9900
Cu2—S2	2.3556 (5)	C10—H10B	0.9900
Cu2—P2	2.2018 (6)	C11—H11A	0.9800
Cu3—Cl2	2.3912 (5)	C11—H11B	0.9800
Cu3—S1	2.4002 (5)	C11—H11C	0.9800
Cu3—S2	2.4939 (5)	C12—C13	1.526 (4)
Cu3—P3	2.1841 (5)	C12—H12A	0.9900
Cu1—Cu3	3.0216 (4)	C12—H12B	0.9900
S1—C1	1.7367 (19)	C13—H13A	0.9800
S2—C1	1.7330 (19)	C13—H13B	0.9800
P1—C10	1.831 (2)	C13—H13C	0.9800
P1—C8	1.836 (2)	C14—C15	1.511 (4)
P1—C6	1.830 (2)	C14—H14A	0.9900
P2—C14	1.816 (2)	C14—H14B	0.9900
P2—C12	1.822 (2)	C15—H15A	0.9800
P2—C16	1.849 (2)	C15—H15B	0.9800
P3—C20	1.830 (2)	C15—H15C	0.9800
P3—C22	1.836 (2)	C16—C17	1.508 (3)
P3—C18	1.842 (2)	C16—H16A	0.9900
N1—C1	1.313 (2)	C16—H16B	0.9900
N1—C5	1.477 (2)	C17—H17A	0.9800
N1—C2	1.480 (2)	C17—H17B	0.9800
C2—C3	1.530 (3)	C17—H17C	0.9800
C2—H2A	0.9900	C18—C19	1.526 (3)
C2—H2B	0.9900	C18—H18A	0.9900
C3—C4	1.527 (3)	C18—H18B	0.9900
C3—H3A	0.9900	C19—H19A	0.9800
C3—H3B	0.9900	C19—H19B	0.9800
C4—C5	1.526 (3)	C19—H19C	0.9800
C4—H4A	0.9900	C20—C21	1.523 (3)
C4—H4B	0.9900	C20—H20A	0.9900
C5—H5A	0.9900	C20—H20B	0.9900
C5—H5B	0.9900	C21—H21A	0.9800
C6—C7	1.515 (4)	C21—H21B	0.9800
C6—H6A	0.9900	C21—H21C	0.9800
C6—H6B	0.9900	C22—C23	1.525 (3)
C7—H7A	0.9800	C22—H22A	0.9900
C7—H7B	0.9800	C22—H22B	0.9900
C7—H7C	0.9800	C23—H23A	0.9800
C8—C9	1.524 (3)	C23—H23B	0.9800
C8—H8A	0.9900	C23—H23C	0.9800
			
Cl1—Cu1—Cl2	96.188 (18)	C9—C8—P1	112.35 (16)
Cl1—Cu1—S1	104.585 (19)	C9—C8—H8A	109.1
Cl1—Cu1—P1	115.51 (2)	P1—C8—H8A	109.1
Cl2—Cu1—S1	100.954 (18)	C9—C8—H8B	109.1
Cl2—Cu1—P1	108.90 (2)	P1—C8—H8B	109.1
S1—Cu1—P1	125.81 (2)	H8A—C8—H8B	107.9
Cl1—Cu2—Cl2	97.080 (18)	C8—C9—H9A	109.5
Cl1—Cu2—S2	106.406 (19)	C8—C9—H9B	109.5
Cl1—Cu2—P2	113.35 (2)	H9A—C9—H9B	109.5
Cl2—Cu2—S2	97.904 (18)	C8—C9—H9C	109.5
Cl2—Cu2—P2	112.82 (2)	H9A—C9—H9C	109.5
S2—Cu2—P2	124.87 (2)	H9B—C9—H9C	109.5
Cl2—Cu3—S1	104.566 (18)	C11—C10—P1	112.60 (17)
Cl2—Cu3—S2	98.030 (18)	C11—C10—H10A	109.1
Cl2—Cu3—P3	118.56 (2)	P1—C10—H10A	109.1
S1—Cu3—S2	74.935 (17)	C11—C10—H10B	109.1
S1—Cu3—P3	127.39 (2)	P1—C10—H10B	109.1
S2—Cu3—P3	123.04 (2)	H10A—C10—H10B	107.8
P1—Cu1—Cu3	132.231 (19)	C10—C11—H11A	109.5
S1—Cu1—Cu3	51.338 (13)	C10—C11—H11B	109.5
Cl1—Cu1—Cu3	109.575 (16)	H11A—C11—H11B	109.5
Cl2—Cu1—Cu3	49.769 (12)	C10—C11—H11C	109.5
P3—Cu3—Cu1	149.44 (2)	H11A—C11—H11C	109.5
Cl2—Cu3—Cu1	55.492 (13)	H11B—C11—H11C	109.5
S1—Cu3—Cu1	49.237 (13)	C13—C12—P2	111.61 (16)
S2—Cu3—Cu1	86.926 (14)	C13—C12—H12A	109.3
Cu1—Cl1—Cu2	81.004 (17)	P2—C12—H12A	109.3
Cu3—Cl2—Cu2	81.010 (16)	C13—C12—H12B	109.3
Cu3—Cl2—Cu1	74.739 (16)	P2—C12—H12B	109.3
Cu2—Cl2—Cu1	73.508 (15)	H12A—C12—H12B	108.0
C1—S1—Cu1	96.15 (6)	C12—C13—H13A	109.5
C1—S1—Cu3	84.76 (6)	C12—C13—H13B	109.5
Cu1—S1—Cu3	79.425 (17)	H13A—C13—H13B	109.5
C1—S2—Cu2	94.21 (6)	C12—C13—H13C	109.5
C1—S2—Cu3	81.97 (6)	H13A—C13—H13C	109.5
Cu2—S2—Cu3	82.517 (17)	H13B—C13—H13C	109.5
C10—P1—C8	102.12 (11)	C15—C14—P2	111.1 (2)
C10—P1—C6	102.40 (12)	C15—C14—H14A	109.4
C8—P1—C6	102.94 (11)	P2—C14—H14A	109.4
C10—P1—Cu1	115.53 (8)	C15—C14—H14B	109.4
C8—P1—Cu1	118.31 (8)	P2—C14—H14B	109.4
C6—P1—Cu1	113.48 (8)	H14A—C14—H14B	108.0
C14—P2—C12	102.29 (12)	C14—C15—H15A	109.5
C14—P2—C16	102.70 (13)	C14—C15—H15B	109.5
C12—P2—C16	101.70 (11)	H15A—C15—H15B	109.5
C14—P2—Cu2	117.20 (8)	C14—C15—H15C	109.5
C12—P2—Cu2	115.53 (8)	H15A—C15—H15C	109.5
C16—P2—Cu2	115.24 (8)	H15B—C15—H15C	109.5
C20—P3—C22	102.17 (10)	C17—C16—P2	112.33 (17)
C20—P3—C18	104.21 (10)	C17—C16—H16A	109.1
C22—P3—C18	101.71 (10)	P2—C16—H16A	109.1
C20—P3—Cu3	114.89 (7)	C17—C16—H16B	109.1
C22—P3—Cu3	113.81 (7)	P2—C16—H16B	109.1
C18—P3—Cu3	118.01 (7)	H16A—C16—H16B	107.9
C1—N1—C5	124.37 (16)	C16—C17—H17A	109.5
C1—N1—C2	123.24 (16)	C16—C17—H17B	109.5
C5—N1—C2	111.41 (15)	H17A—C17—H17B	109.5
N1—C1—S2	121.62 (14)	C16—C17—H17C	109.5
N1—C1—S1	120.11 (14)	H17A—C17—H17C	109.5
S2—C1—S1	118.25 (11)	H17B—C17—H17C	109.5
N1—C2—C3	103.77 (16)	C19—C18—P3	114.33 (14)
N1—C2—H2A	111.0	C19—C18—H18A	108.7
C3—C2—H2A	111.0	P3—C18—H18A	108.7
N1—C2—H2B	111.0	C19—C18—H18B	108.7
C3—C2—H2B	111.0	P3—C18—H18B	108.7
H2A—C2—H2B	109.0	H18A—C18—H18B	107.6
C4—C3—C2	103.25 (15)	C18—C19—H19A	109.5
C4—C3—H3A	111.1	C18—C19—H19B	109.5
C2—C3—H3A	111.1	H19A—C19—H19B	109.5
C4—C3—H3B	111.1	C18—C19—H19C	109.5
C2—C3—H3B	111.1	H19A—C19—H19C	109.5
H3A—C3—H3B	109.1	H19B—C19—H19C	109.5
C5—C4—C3	103.30 (16)	C21—C20—P3	113.07 (16)
C5—C4—H4A	111.1	C21—C20—H20A	109.0
C3—C4—H4A	111.1	P3—C20—H20A	109.0
C5—C4—H4B	111.1	C21—C20—H20B	109.0
C3—C4—H4B	111.1	P3—C20—H20B	109.0
H4A—C4—H4B	109.1	H20A—C20—H20B	107.8
N1—C5—C4	102.84 (16)	C20—C21—H21A	109.5
N1—C5—H5A	111.2	C20—C21—H21B	109.5
C4—C5—H5A	111.2	H21A—C21—H21B	109.5
N1—C5—H5B	111.2	C20—C21—H21C	109.5
C4—C5—H5B	111.2	H21A—C21—H21C	109.5
H5A—C5—H5B	109.1	H21B—C21—H21C	109.5
C7—C6—P1	113.13 (18)	C23—C22—P3	112.66 (16)
C7—C6—H6A	109.0	C23—C22—H22A	109.1
P1—C6—H6A	109.0	P3—C22—H22A	109.1
C7—C6—H6B	109.0	C23—C22—H22B	109.1
P1—C6—H6B	109.0	P3—C22—H22B	109.1
H6A—C6—H6B	107.8	H22A—C22—H22B	107.8
C6—C7—H7A	109.5	C22—C23—H23A	109.5
C6—C7—H7B	109.5	C22—C23—H23B	109.5
H7A—C7—H7B	109.5	H23A—C23—H23B	109.5
C6—C7—H7C	109.5	C22—C23—H23C	109.5
H7A—C7—H7C	109.5	H23A—C23—H23C	109.5
H7B—C7—H7C	109.5	H23B—C23—H23C	109.5
			
C5—N1—C1—S2	6.0 (2)	C6—P1—C8—C9	−176.38 (18)
C2—N1—C1—S2	173.70 (13)	Cu1—P1—C8—C9	−50.4 (2)
C5—N1—C1—S1	−172.23 (14)	C8—P1—C10—C11	−176.76 (19)
C2—N1—C1—S1	−4.5 (2)	C6—P1—C10—C11	76.9 (2)
Cu2—S2—C1—N1	−93.58 (15)	Cu1—P1—C10—C11	−47.0 (2)
Cu3—S2—C1—N1	−175.40 (15)	C14—P2—C12—C13	178.71 (19)
Cu2—S2—C1—S1	84.66 (10)	C16—P2—C12—C13	−75.3 (2)
Cu3—S2—C1—S1	2.84 (9)	Cu2—P2—C12—C13	50.2 (2)
Cu1—S1—C1—N1	96.59 (14)	C12—P2—C14—C15	−81.4 (2)
Cu3—S1—C1—N1	175.33 (15)	C16—P2—C14—C15	173.45 (19)
Cu1—S1—C1—S2	−81.67 (10)	Cu2—P2—C14—C15	46.1 (2)
Cu3—S1—C1—S2	−2.93 (9)	C14—P2—C16—C17	−84.3 (2)
C1—N1—C2—C3	−176.54 (16)	C12—P2—C16—C17	170.03 (19)
C5—N1—C2—C3	−7.4 (2)	Cu2—P2—C16—C17	44.3 (2)
N1—C2—C3—C4	28.69 (19)	C20—P3—C18—C19	−91.44 (17)
C2—C3—C4—C5	−39.4 (2)	C22—P3—C18—C19	162.62 (16)
C1—N1—C5—C4	152.09 (17)	Cu3—P3—C18—C19	37.36 (18)
C2—N1—C5—C4	−16.9 (2)	C22—P3—C20—C21	179.63 (16)
C3—C4—C5—N1	34.37 (19)	C18—P3—C20—C21	74.03 (18)
C10—P1—C6—C7	−178.43 (18)	Cu3—P3—C20—C21	−56.63 (18)
C8—P1—C6—C7	75.85 (19)	C20—P3—C22—C23	73.77 (18)
Cu1—P1—C6—C7	−53.22 (19)	C18—P3—C22—C23	−178.70 (16)
C10—P1—C8—C9	77.7 (2)	Cu3—P3—C22—C23	−50.70 (18)

### Hydrogen-bond geometry (Å, º)

Cg1 is the centroid of the (Cu,S1,S2,C1) chelate ring.

**Table:**

*D*—H···*A*	*D*—H	H···*A*	*D*···*A*	*D*—H···*A*
C20—H20*B*···Cl1^i^	0.99	2.81	3.722 (2)	154
C22—H22*B*···Cl1^i^	0.99	2.80	3.720 (2)	154
C3—H3*B*···*Cg*1^ii^	0.99	2.83	3.705 (2)	148

Symmetry codes: (i) *x*, *y*, *z*−1; (ii) −*x*+1, −*y*+1, −*z*+1.
